# The Playing Brain. The Impact of Video Games on Cognition and Behavior in Pediatric Age at the Time of Lockdown: A Systematic Review

**DOI:** 10.3390/pediatric13030047

**Published:** 2021-07-14

**Authors:** Daniela Smirni, Elide Garufo, Luca Di Falco, Gioacchino Lavanco

**Affiliations:** Department of Psychology, Educational Science and Human Movement, University of Palermo, 90128 Palermo, Italy; elidegarufo@gmail.com (E.G.); lucadifalco92@gmail.com (L.D.F.); gioacchino.lavanco@unipa.it (G.L.)

**Keywords:** children, adolescents, playing video games, addiction behavior, cognitive skills, COVID-19 lockdown

## Abstract

A growing number of children and adolescents play video games (VGs) for long amounts of time. The current outbreak of the Coronavirus pandemic has significantly reduced outdoor activities and direct interpersonal relationships. Therefore, a higher use of VGs can become the response to stress and fear of illness. VGs and their practical, academic, vocational and educational implications have become an issue of increasing interest for scholars, parents, teachers, pediatricians and youth public policy makers. The current systematic review aims to identify, in recent literature, the most relevant problems of the complex issue of playing VGs in children and adolescents in order to provide suggestions for the correct management of VG practice. The method used searches through standardized search operators using keywords related to video games and the link with cognition, cognitive control and behaviors adopted during the pandemic. Ninety-nine studies were reviewed and included, whereas twelve studies were excluded because they were educationally irrelevant. Any debate on the effectiveness of VGs cannot refer to a dichotomous approach, according to which VGs are rigidly ‘good’ or ‘bad’. VGs should be approached in terms of complexity and differentiated by multiple dimensions interacting with each other.

## 1. Introduction

In the last decades, a very large body of literature has shown an increasing interest in video games (VGs) and their impact on the brain, cognition and behavior, especially in children and adolescents [1]. Indeed, a widely growing number of children and adolescents play VGs for a long time, often developing real addictive behaviors [2,3]. In addition, the current outbreak of the COVID-19 pandemic and the following lockdown have significantly reduced outdoor activities and direct interpersonal relationships [4,5]. However, literature data are still inconsistent. For example, according to some meta-analytic reviews [6,7,8], exposure to violent VGs is a causal risk factor for increased aggressive behavior, cognition and affection in children and adolescents. Conversely, many cross-sectional and intervention studies have shown that the intensive use of some types of VGs leads to significant improvements in many cognitive domains and behaviours [1,9,10,11]. Video games are even considered as ‘virtual teachers’ and effective and ‘exemplary teachers’ [12,13].

The current systematic review focuses on some crucial outstanding issues within the debate on the effects of VGs on cognition and behavior in order to provide suggestions for parents, pediatricians, health providers and educators dealing with pediatric ages, especially in the complex pandemic period. Namely, it analyzes the most debated and educationally relevant problems on the relationship between video games, cognition and behavior: 1. video games’ effects on cognitive function; 2. video games’ effects on attention and addictive behaviors; 3. video games and prosocial or aggressive behavior. Therefore, the current analysis may be accounted as an original contribution to the practical dimension in the educational and rehabilitation field for parents and educators.

Early common predominant opinions mainly focused on VGs according to dichotomous thinking, as enjoyable entertainment or harmful tools [14]. The recent literature instead provided evidence on the impact of VGs on the brain and its functional modifications while playing [15,16,17,18,19], showing that video games involve different cortical and subcortical structures, with cognitive and emotional competence, such as frontal and prefrontal regions, the posterior and superior parietal lobe, the anterior and posterior cingulate cortices, limbic areas, the amygdala, the entorhinal cortex and basal nuclei [1,20,21,22]. 

Mondéjar and colleagues [15], in a group of twelve healthy preadolescents between 8 and 12 years old, evaluated the frontal lobe activity and the different types of cognitive processing during five platform-based action videogame mechanics: 1. accurate action, related to processes such as concentration, attention, impulse control and information comprehension; 2. timely action, related to working memory, selective attention, decision-making, problem solving and perception; 3. mimic sequence, related to working memory, focalized attention and inhibition control; 4. pattern learning, as selective attention, planning, inhibition control and spatial orientation; 5. logical puzzles related to attention, working memory, the capacity for abstraction, information processing, problem solving, or resistance to interference. They found prominent bioelectrical prefrontal activity during the performance related to executive functions (timely action, pattern learning, logical puzzles) and more global brain activity and a higher presence of alpha waves, or a greater activation of the temporal lobe, in the accurate action and mimic sequence. Similarly, they correlated higher magnitudes on frequency bands with five game mechanics in ten healthy children, who played with a VG platform for an average of about 20 min [16]. Theta waves, related to memory and emotions, were more significant in the five mechanics, while beta waves, related to concentration, were more prominent in only two. Moreover, activation was more significant in the intermediate and occipital areas for all the mechanics, while recurrent magnitude patterns were identified in three mechanics.

Similarly, Lee et al. [17], found a thinner cortex and a smaller gray matter volume in critical areas for evaluating reward values, error processing and adjusting behavior, namely, the anterior cingulate cortex, the orbitofrontal cortex and the frontoparietal areas, in young male adults with internet gaming disorders, compared to age-matched healthy male controls. A neuroimaging study examined in individuals affected by gaming disorders the differences during the playing of a violence-related vs. a non-violence-related version of the same VG [18]. While functional connectivity of the reward-related network and the behavioral inhibition system was altered, the orbitofrontal cortex and anterior cingulate cerebral area were overstimulated, similarly to smart drug addiction [17,23].

Recently, Kwak et al. [19] longitudinally compared 14 adolescents with internet gaming disorder to 12 professional internet gaming students who practiced for about ten hours a day, within a defined support system that included practice, physical exercise, lectures on team strategy, rest and mealtimes. After one year, both groups showed increased brain activity within the attention system of the parietal lobe. However, professional gamers improved problematic behaviors, impulsivity, aggression, depression and anxiety, while adolescents with internet gaming disorder showed no behavioral improvement and a dysfunctional brain activity within the impulse control network in the left orbitofrontal cortex.

## 2. Methods

The current systematic review was structured according to the guidelines and recommendations contained in the PRISMA statement [24].

**Eligibility Criteria**

Both experimental and correlational studies and meta-analyses between the years of 2000 and 2020 that investigated outcomes of VG exposure were included. They were considered children and adolescents. Studies employing different methodologies were included: studies in which naive participants were trained to use a VG versus a control group and studies comparing experienced versus non-gamers, or inexperienced players. Primary outcome measures were any type of structural and functional data obtained using neuroimaging techniques and behavioral testing.

**Information Sources**

One hundred and twenty-two studies were identified through electronic database searching in Ovid MEDLINE, Embase, PsycINFO, PubMed, Scopus (Elsevier) and Web of Sciences. The final database search was run on January 2021 using the following keywords: video games; video games and cognition; video games and epidemic; cognitive control; behavior control; brain and video games; spatial cognition; prosocial behavior; violence in video games; aggressive behavior; addictions in adolescents; children and video games. 

**Study Selection**

Inclusion criteria: written in English; published since 2000; deals in depth with cognitive skills, attention, executive functions, or cognitive control; follows a high methodological rigor.

Exclusion criteria: does not refer to key topics directly; the full text could not be obtained; lack of transparency due to missing methodology information. Ninety-nine studies were reviewed and included, whereas twelve studies were excluded because they were irrelevant to the topic or because the full text was not obtained. General communication materials, such as pamphlets, posters and infographics, were excluded as they do not provide evidence about their effectiveness.

**Figure 1:**
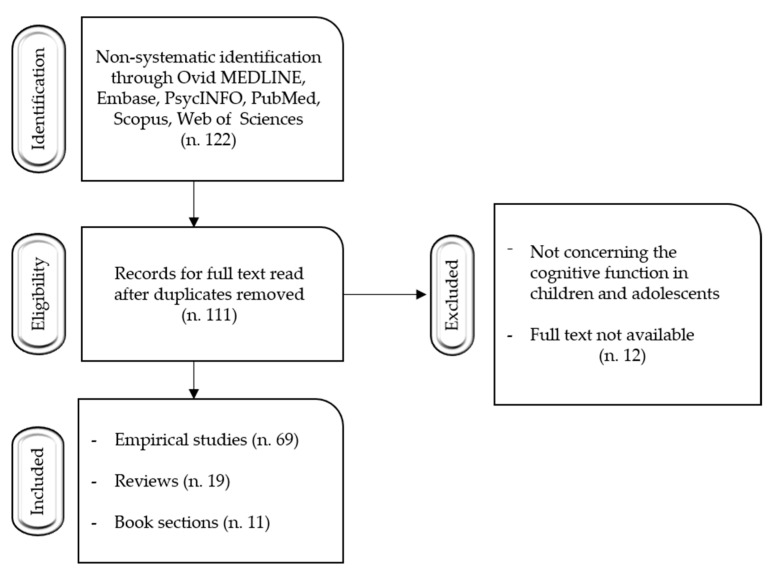
Selection of studies flowchart.

Figure 1 shows the selection of studies flowchart.

## 3. Results

### 3.1. Effect of Video Games on Cognitive Functions

Any modern VG requires an extensive repertoire of attentional, perceptual and executive abilities, such as a deep perceptual analysis of complex unfamiliar environments, detecting relevant or irrelevant stimuli, interference control, speed of information processing, planning and decision making, cognitive flexibility and working memory. 

Literature data in the last years have proven that VGs may improve a variety of cognitive domains [1,25] as, for example, even just 10 hours of VG could improve spatial attention and mental rotation [26,27]. A large variety of design studies reported in habitual players better performance in multiple cognitive domains, including selective attention [3,21,26,28], speed of processing [21,28], executive functions [29,30] and working memory [31]. Similarly, a large body of intervention studies have shown improvements in the same cognitive domains in non-players following training in action VGs [27,32,33,34,35,36,37]. Recently, Benoit et al. [38] examined in 14 professional VG players and 16 casual VG players various cognitive abilities, such as processing speed, attention, memory, executive functions, manual dexterity and tracking multiple objects in three dimensions [39]. Professional players showed a very large advantage in visual–spatial short-term memory and visual attention, and less in selective and sustained attention and auditory working memory. Moreover, they showed better speed thresholds in tracking multiple objects in three dimensions overall, though the rate of improvement did not differ in the two groups. In two previous meta-analyses, Bediou et al. [40] focused on the long-term effects of action VGs on various cognitive domains using both cross-sectional and intervention studies. Overall, the results documented a positive impact of action video gaming on cognition. In cross-sectional studies, a main effect of about half a standard deviation was found. The habitual action game players showed better performance than non-players. Likewise, intervention studies showed about a third of a standard deviation advantage in cognition domains in action VG trainees. Perception, spatial cognition and top-down attention were the three cognitive domains with the most robust impact [40]. 

Homer et al. [41] examined the effectiveness of a custom-designed VG (‘alien game’) in a group of 82 healthy adolescents (age range 14–18 years; average = 15.5 years) trained to play for 20 min per week for 6 consecutive weeks. Such a digital game was devised to target, in a fun way, the specific executive ability of shifting, as the ability to shift between tasks or mental sets, hypothesizing that after playing the ‘alien game’ over a period of several weeks, adolescents would show significant improvements in the targeted ability. Pre- and post-test measures of another executive ability, inhibition, as the ability to control a prepotent response, were also recorded in order to examine the extent to which training would transfer from one executive ability to another. Significant advantages both in shifting and in inhibition abilities were found, providing evidence that VGs can be effective tools for training executive abilities [42,43].

Similarly, Oei and Patterson [44] examined the effect of action and non-action VGs on executive functions. Fifty-two non-VG gamers played one of four different games for 20 h. Pre- and post-training tests of executive function were administered. The group that trained on the physics-based puzzle game, demanding high level planning, problem solving, reframing, strategizing and new strategies from level to level, improved in several aspects of executive function. In a previous study, the same authors [45] instructed 75 non-gamers, (average age 21.07 ± 2.12) to play for 20 h, one hour a day/five days a week over four weeks. They compared effects of action and non-action games to examine whether non-action games also improve cognition. Four tests pre- and post-training were administered. The results showed that cognitive improvements were not limited to training with action games and that different games improved different aspects of cognition. Action VGs have even been used to treat dyslexic children [46,47]. Only 12 h of action VGs, for nine sessions of 80 min per day, significantly improved reading and attentional skills [48].

Moreover, several meta-analytic studies provide evidence that action VG training may become an efficient way to improve the cognitive performance of healthy adults. Wang et al. [49], in a meta-analysis, found that healthy adults achieve moderate benefits from action VG training in overall cognitive ability and moderate to small benefits in specific cognitive domains. In contrast, young adults gain more benefits than older adults in both overall cognition and specific cognitive domains. 

In summation, the studies on VG effects, by different methodologies, document both in adults and in children significant positive outcomes in different cognitive domains. Such performance improvements may be paralleled by functional brain remodelling [14].

### 3.2. Video Games Effect on Attention and Addictive Behaviors

Attentional problems are accounted as a crucial area of focus on outcomes of intensive game-play practices in children and adolescents. However, literature on the topic appears inconsistent. While some research has found mixed results [50] or a positive effect [51,52,53], or no relationship between VG practice and attention, other studies have linked VG playing with greater attention problems, such as impulsiveness, self-control, executive functioning, and cognitive control [53,54,55]. 

Gentile et al. [56], examining longitudinally, over 3 years, a large sample of child and adolescent VG players aged 8–17 (mean = 11.2 ± 2.1), suggested a bidirectional causality: children who spend more time playing VGs have more attention problems; in turn, subjects who have more attention problems spend more time playing VGs. Therefore, children and adolescents with attention problems are more attracted to VGs (excitement hypothesis), and, in turn, they find it less engaging to focus on activities requiring more control and sustained attention, such as educational activities, homework or household chores (displacement hypothesis). According to such hypotheses, and to the operant conditioning model [57,58], VGs, providing strong motivational cues, become more rewarding for impulsive children and teenagers [51] who, in such contexts, experience a sense of value and feelings of mastery that they do not experience in their daily relationships [59].

Actually, any modern VG is a highly engaging activity with a variety of attractive cues, such as, for example, violence, rapid movement, fast pacing and flashing lights [60,61]. According to the attractive hypothesis [56], it may provide a strong motivation and support for attention and even become addictive, especially in subjects with problems maintaining attention in usual, monotonous and poorly engaging tasks. Therefore, paradoxically, a greater VG exposure may improve visual attention skills involved in such engaging play [26], but it may impair the ability to selectively focus on a target for lasting time, without external exciting cues.

Probably, in line with the bidirectional causality framework [56], such rewarding conditions could become the psychological context for the structuring of addictive behaviors, such as a sense of euphoria while playing, feeling depressed away from the game, an uncontrollable and persistent craving to play, neglect of family and friends, problems with school or jobs, alteration of sleeping routines, irregular meals and poor hygiene [14]. The most psychologically fragile subjects may be most attracted to an engaging and rewarding activity, ensuring an effective compensation to their fragility [14]. However, the topic of video game addiction continues to present today many outstanding issues. There is a large consensus that ‘pathological use’ is more debilitating than ‘excessive use’ of VGs alone [62,63,64]. Addictive behavior appears associated with an actual lowering in academic, social, occupational, developmental and behavioral dimensions, while excessive use may simply be an excessive amount of time gaming. According to Griffiths’ suggestions, ‘healthy excessive enthusiasms add to life, whereas addiction takes away from it’ [65]. However, it is sometimes difficult to identify the clear line between unproblematic overuse of gaming and the pathological and compulsive overuse that compromises one’s lifestyle and psychosocial adjustment [66,67,68]. Therefore, there may be a risk of stigmatizing an enjoyable practice, which, for a minority of excessive users, may be associated with addiction-related behaviors [69,70]. Przybylski and colleagues, in four survey studies with large international cohorts (N = 18,932), found that the percentage of the general population who could qualify for internet gaming disorders was extremely small (less than one percent) [71].

In such a discussion of the pathological nature of VGs, another outstanding question is whether pathological play is a major problem, or if it is the phenomenological manifestation of another pathological condition. Several studies have suggested that video game play can become harmful enough to be categorized as a psychiatric disorder, or it could be a symptom of an underlying psychopathological condition, such as depression or anxiety. Moreover, the functional impairments observed in individuals with game addictions are also thought to be similar to the impairments observed in other addictions. Neuroimaging studies have shown that the brain reward pathways which are activated during video game playing are also activated during cue-induced cravings of drug, alcohol or other type of substances abuse [72,73,74].

Some longitudinal studies [14,75,76] proved that pathological addictive behaviors, such as depression, are likely to be outcomes of pathological gaming rather than predictors of it [77,78]. Lam and Peng [79], in a prospective study with a randomly generated cohort of 881 healthy adolescents aged between 13 and 16 years, found that the pathological use of the internet results in later depression. Similarly, Liau et al. [80], in a 2-year longitudinal study involving 3034 children and adolescents aged 8 to 14 years, found that pathological video gaming has potentially serious mental health consequences, in particular of depression. 

In summary, attention problems and addictive behaviors in the context of VGs should be addressed in a circular and bidirectional way in which each variable can influence the others.

### 3.3. Video Games Effect and Prosocial and Aggressive Behaviors 

The positive impact of video games also concerns the social and relational dimension, as occurs in the VG training of prosocial or educational skills. Several studies have reported that playing prosocial VGs, even for a short time, increases prosocial cognition [81], positive affect [82] and helping behaviors [13,81,82,83,84,85], whereas it decreases antisocial thoughts and the hostile expectation bias, such as the tendency to perceive any provocative actions of other people as hostile even when they are accidental [13,86]. Such findings have been found in correlational, longitudinal and experimental investigations [82,85,87].

In four different experiments [13], playing VGs with prosocial content was positively related to increased prosocial behavior, even though participants played the VGs for a relatively short time, suggesting that VGs with prosocial content could be used to improve social interactions, increase prosocial behavior, reduce aggression and encourage tolerance. 

Following experimental, correlational, longitudinal and meta-analytic studies provided further evidence that playing a prosocial VG results in greater interpersonal empathy, cooperation and sharing and subsequently in prosocial behavior [87,88,89,90].

Such literature’s data are consistent with the General Learning Model [91,92], according to which the positive or negative content of the game impacts on the player’s cognition, emotions and physiological arousal, which, in turn, leads to positive or negative learning and behavioral responses [12,93,94,95]. Therefore, repeated prosocial behavioral scripts can be translated into long-term effects in cognitive, emotional and affective constructs related to prosocial actions, cognition, feelings, and physiological arousal, such as perceptual and expectation schemata, beliefs, scripts, attitudes and stereotypes, empathy and personality structure [83,91].

In the same conceptual framework, educational video games have been found to positively affect behaviors in a wide range of domains [12], school subjects [96] and health conditions [97,98]. In randomized clinical trials, for example, diabetic or asthmatic children and adolescents improved their self-care and reduced their emergency clinical utilization after playing health education and disease management VGs. After six months of playing, diabetic patients decreased their emergency visits by 77 percent [99]. Therefore, well-designed games can provide powerful interactive experiences that can foster young children’s learning, skill building, self-care and healthy development [100]. 

Violence in VGs is a matter of intense debate, both in public opinion and in the scientific context [101,102]. A vast majority of common opinions, parents and educators consider the violence of VGs as the most negatively impacting feature to emotional and relational development of youth and children. Actually, studies agree on the negative impact of violent video games on aggressive behavior. Several meta-analyses have examined violent VGs [6,7,8,103] and, although they vary greatly in terms of how many studies they include, they seem to agree with each other. The most comprehensive [8] showed that violent VGs, gradually and unconsciously, as a result of repeated exposure to justified and fun violence, would increase aggressive thoughts, affect and behavior, physiological persistent alertnes, and would desensitize players to violence and to the pain and suffering of others, supporting a perceptual and cognitive bias to attribute hostile intentions to others. 

Similarly, experimental, correlational and longitudinal studies supported the causal relationship between violent VGs and aggression, in the short- and long-term, both in a laboratory and in a real-life context. A greater amount of violent VGs, or even a brief exposure, were significantly associated with more positive attitudes toward violence [104], higher trait hostility [105] and with increased aggressive behaviors [106], physical fights [107] and aggressive thoughts [108] and affect [109]. In a two-year longitudinal study, children and adolescents who played a lot of violent VGs showed over time more aggressive behaviors, including fights and delinquency [110]. Saleem, Anderson and Gentile [82] examined the effects of short-term exposure to prosocial, neutral and violent VGs in a sample of 191 children of 9–14 years old. Results indicated that while playing prosocial games increased helpful and decreased hurtful behaviour, the violent games had the opposite effect.

In summation, the overall literature data support the opinion that violent video games, over time, affect the brain and activate a greater availability to aggressive behavior patterns, although some researchers have pointed out that the negative effects of violent VGs are small and may be a publication bias [14,111]. 

## 4. Discussion

The focus of the current overview was to identify, from a functional point of view, the most significant issues in the debate on the impact of VGs on cognition and behavior in children and adolescents, in order to provide suggestions for a proper management of VG practice. 

Overall, the reviewed literature agrees in considering the practice of VGs as much more than just entertainment or a leisure activity. Moreover, research agrees that any debate on the effectiveness of VGs cannot refer to a unitary construct [14], nor to a rigidly dichotomous approach, according to which VGs are ‘good’ or ‘bad’ [1,12,112,113]. 

The term ‘video game’ should be viewed as an ‘umbrella term’ that covers different meanings, far from a single unitary construct [14,114]. Furthermore, VGs and their effects should be approached in terms of complexity and differentiated by multiple dimensions interacting with each other and with a set of other variables, such as, for example, the player’s age and personality traits, the amount of time spent playing, the presence of an adult, the game alone or together with others and so on [115]. 

Gentile and colleagues [116,117,118,119] have identified five main features of VGs that can affect players: 1. amount of play; 2. content; 3. context; 4. structure; and 5. mechanics. Each of these aspects can produce or increase different thoughts, feelings and behaviors.

However, the content effects, individually focused, are frequently overemphasized. According to the General Learning Model, children would learn the contents of the specific games and apply them to their lives. Nevertheless, a violent game using a team-based game modality may have different impacts than a violent game using a ‘free for all’ game modality. Although both are equally violent games, the former could suggest teamwork and collaborative behaviors, while playing in an ‘everyone for oneself’ mode could foster less empathy and more aggressive thoughts and behaviors [8,88]. 

Likewise, the outside social context can have different effects and it may even mitigate or reinforce the effects of the content. Playing violent games together with others could increase aggression outcomes if players reinforce each other in aggressive behavior. Instead, it could have a prosocial effect if the motivations to play together are to help each other [120]. 

According to the dominant literature, the psychological appeal of video games may be related to an operant conditioning that reinforces multiple psychological instances, including the need for belonging and social interaction [57,58]. On such drives and reinforcements, the playing time can expand, and it may become endless in addicted subjects. However, the amount of play, regardless of the content, can become harmful when it displaces beneficial activities, affects academic performance or social dimensions [52,121], or supports health problems, such as, for instance, obesity [122,123,124], repetitive strain disorder and video game addiction [76,83]. However, a greater amount of time inevitably implies increased repetition of other game dimensions. Therefore, it is likely that some associations between time spent and negative outcomes result from other dimensions, and not from amount of time per se. Moreover, children who perform poorly at school are likely to spend more time playing games, according to the displacement hypothesis, but over time, the excessive amount of play may further damage academic performance in a vicious circle [116]. 

VGs can also have a different psychological appeal in relation to their structural organization and the way they are displayed. Many structural features can affect playing behavior, regardless of the individual’s psychological, physiological, or socioeconomic status [125], such as, for instance, the degree of realism of the graphics, sound and back-ground, the game duration, the advancement rate, the game dynamics such as exploring new areas, elements of surprise, fulfilling a request, the control options of the sound, graphics, the character development over time and character customization options, the winning and losing features as the potential to lose or accumulate points, finding bonuses, having to start a level again, the ability to save regularly, the multi-player option building alliances and beating other players [125]. 

The more or less realistic mechanics can also configure the game differently and affect fine or gross motor skills, hand-eye coordination or even balance skills, depending on the type of controller, such as a mouse and keyboard, a game control pad, a balance board, or a joystick.

Therefore, VGs may differ widely in multiple dimensions and, as a result, in their effects on cognitive skills and behavior [3,33]. Moreover, the different dimensions may interact with each other and with the psychological, emotional and personality characteristics of the individual player and context. Even the same game can have both positive and negative effects in different contexts and for different subjects. 

The current analysis of the literature, therefore, supports the need for further experimental and longitudinal research on the role of multiple characteristics of video games and their interactions. A wide-ranging approach dynamically focused on the multiple dimensions will allow a deeper theoretical understanding of the different aspects of video games.

Nevertheless, according to common opinion, the violence would always have a negative impact on behavior, especially in pediatric subjects. However, a strictly causal relationship between violent VGs and aggressive behavior appears rather reductive [126,127]. Aggressive behavior is a complex one and arises from the interaction of a lot of factors. Therefore, violent VGs, with no other risk factors, should not be considered ‘per se’ the linear cause and single source of aggressive or violent behavior. Antisocial outcomes can be influenced by personality variables, such as trait aggression, or by a number of the ‘third variables’ such as gender, parental education, exposure to family violence and delinquency history [83]. According to social learning theories [128], aggressive behavior would arise from repeated exposure to violence patterns [129]. Therefore, children who have other risk factors for violent or aggressive behavior, such as violent family patterns, excessive amount of time spent playing, playing alone, and so on are more likely to have negative consequences from playing violent video games.

An alternative theoretical framework [126,127] assumes that violent behavior would result from the interaction of genetically predisposed personality traits and stressful situations. In such a model, violent VGs would act as ‘stylistic catalysts’ [127], providing an individual predisposed to violence with the various models of violent behavior. Therefore, an aggressive child temperament would derive from a biological pathway, while the violent VG, as a ‘stylistic catalyst’, may suggest the specific violent behavior to enact. 

Conversely, playing prosocial VGs, even for a short time, increases prosocial cognition, affect and behaviors in children and adolescents [13,81,82,83,84,85,89]. Several intervention or training studies showed that a prosocial VG should activate experiences, knowledge, feelings and patterns of behavior relating to prosocial actions, cognition, feelings and physiological arousal. In turn, in line with the General Learning Model, [91,130], recurrent prosocial behavioral scripts produce new learning, new behavioral patterns and emotional and affective cognitive constructs [83]. 

Moreover, several studies emphasize the educational and academic potential of VGs that may become effective and ‘exemplary teachers’ [12,82] providing fun and motivating contexts for deep learning in a wide range of content [12], such as school learning [96], rehabilitation activities [46,47], new health care and protection behavior development and the enhancement of specific skills [97,99,100]. Similarly, the literature data document that the intensive use of VGs results in generalized improvements in cognitive functions or specific cognitive domains, and in behavioral changes [1]. Actually, VGs involve a wide range of cognitive functions, and attentional, perceptual, executive, planning and problem solving skills. They can, therefore, be expected to improve different perceptual and cognitive domains. However, on a methodological level, the impact on behavior and cognition cannot be simplistically viewed as the linear result of a causal relationship between VG and performance. For instance, subjects with better perceptual abilities are likely to choose to play and, as a result, their increase in performance may reflect their baseline level rather than the effects of the game.

Studies focused on the attentional functions in VG playing reported inconsistent data. Playing action games may improve attention skills implied in a specific game. However, according to the attractive hypothesis [56] and operant conditioning theory, children and adolescents with attentional problems may be attracted by the motivating and engaging VG activities. On the other hand, children and adolescents with a wider VG exposure show greater attention problems [53]. The relationship between VGs and attention, then, seem to be approached in terms of bidirectional causality [56]. 

Similarly, since VGs and their cues appear more pleasant and desirable, a large amount of attractive VG exposure can lead to addiction and impair ability to focus on effortful goal oriented behavior [131]. However, the literature does not yet appear to agree on the objective diagnostic criteria for classifying behavioral game addiction [132].

In the fifth edition appendix of the Diagnostic and Statistical Manual of Mental Disorders [133], the diagnostic criteria for Internet Gaming Disorder included both specific internet games and offline games. However, this has led to some confusion as to whether excessive video games must necessarily occur online [134,135]. According to some authors, since ‘Internet addiction’ includes heterogeneous behaviors and etiological mechanisms, the term ‘video game disorder’ or simply ‘gaming disorder’ would be more suitable [136,137], while the term ‘Internet addiction’ appears inappropriate. Individuals rarely become addicted to the medium of the internet itself [137,138]. Moreover, it has also been supported theoretically [135] and empirically proven [139] that problematic internet use and problematic online gaming are not the same.

The debate on the relationship between pure game addiction behaviors and game addiction in comorbidity with other psychiatric disorders appears still on. Some researchers have argued that game addiction, as a standalone clinical entity, does not exist [140], but it is simply a symptom of psychiatric illnesses such as major depressive disorder or Attention Deficit Hyperactivity Disorder. Equally poorly defined is the question of genetic predisposition and vulnerability to game addiction.

Likewise, the relationship between clinical symptoms and changes in brain activity and the dynamics by which video games triggers such widespread brain plasticity needs to be more clearly defined. 

## 5. Conclusions

The current analysis of the literature provides strong evidence on the power of video games as highly motivating and engaging tools in the broader context of cognitive, emotional and relational development of children and adolescents. However, the effectiveness of such tools does not arise exclusively from their content, but it results from a set of variables interacting each other. 

Video games, beyond their content, can favor pathological aggression, withdrawal, escape from reality and reduction of interests. Virtual reality becomes more attractive than the real one and can become the ‘non-place’ to escape from the complexity of everyday life. Recently, to contain the spread of the COVID-19 pandemic, health authorities have forced populations to stay home and children and adolescents may experience an exacerbation of exposure to video games. 

Parents, educators and teachers should ensure an educational presence, monitoring times and modalities of VG practice in a broader context in which children and adolescents live with a wider repertoire of interests, without losing social and relational engagement. Moreover, pediatric health care visits may be a great opportunity to support parents helping children to deal with media and video games. 

On these assumptions, as practical suggestions to prevent or mitigate addictive behaviors, parents and educators should enforce the golden rule as the educational presence of the adult. 

Moreover, in line with the literature, the core values to prevent a negative impact of video games should be focused on a few rules to be proposed with assertiveness and authority: 1. set a clear time limit to play, 2. prefer games that can also be played with family, 3. alternate video games with other games and activities, 4. avoid highly addictive games, 5. keep a social life in the real world.

## Data Availability

Data sharing not applicable.

## References

[1] Palaus M., Marron E.M., Viejo-Sobera R., Redolar-Ripoll D. (2017). Neural basis of video gaming: A systematic review. Front. Hum. Neurosci..

[2] Mayer R.E. (2014). Incorporating motivation into multimedia learning. Learn. Instr..

[3] Green C.S., Bavelier D. (2012). Learning, attentional control, and action video games. Curr. Biol..

[4] Smirni P., Lavanco G., Smirni D. (2020). Anxiety in Older Adolescents at the Time of COVID-19. J. Clin. Med..

[5] Smirni D. (2021). Noli Timere: The Role of Reassuring Adults in Dealing with COVID-19 Anxiety in Pediatric Age. Pediatr. Rep..

[6] Anderson C.A., Bushman B.J. (2001). Effects of violent video games on aggressive behavior, aggressive cognition, aggressive affect, physiological arousal, and prosocial behavior: A meta-analytic review of the scientific literature. Psychol. Sci..

[7] Anderson C.A. (2004). An update on the effects of playing violent video games. J. Adolesc..

[8] Anderson C.A., Shibuya A., Ihori N., Swing E.L., Bushman B.J., Sakamoto A., Rothstein H.R., Saleem M. (2010). Violent video game effects on aggression, empathy, and prosocial behavior in eastern and western countries: A meta-analytic review. Psychol. Bull..

[9] Powers K.L., Brooks P.J., Aldrich N.J., Palladino M.A., Alfieri L. (2013). Effects of video-game play on information processing: A meta-analytic investigation. Psychon. Bull. Rev..

[10] Lampit A., Hallock H., Valenzuela M. (2014). Computerized cognitive training in cognitively healthy older adults: A systematic review and meta-analysis of effect modifiers. PLoS Med..

[11] Toril P., Reales J.M., Ballesteros S. (2014). Video game training enhances cognition of older adults: A meta-analytic study. Psychol. Aging.

[12] Gentile D.A., Gentile J.R. (2008). Violent video games as exemplary teachers: A conceptual analysis. J. Youth Adolesc..

[13] Greitemeyer T., Osswald S. (2010). Effects of prosocial video games on prosocial behavior. J. Pers. Soc. Psychol..

[14] Bavelier D., Green C.S., Han D.H., Renshaw P.F., Merzenich M.M., Gentile D.A. (2011). Brains on video games. Nat. Rev. Neurosci..

[15] Mondéjar T., Hervas R., Johnson E., Gutierrez C., Latorre J.M. (2016). Correlation between videogame mechanics and executive functions through EEG analysis. J. Biomed. Inform..

[16] Mondéjar T., Hervás R., Johnson E., Gutiérrez-López-Franca C., Latorre J.M. (2019). Analyzing EEG waves to support the design of serious games for cognitive training. J. Ambient Intell. Humaniz. Comput..

[17] Lee D., Park J., Namkoong K., Kim I.Y., Jung Y.-C. (2018). Gray matter differences in the anterior cingulate and orbitofrontal cortex of young adults with Internet gaming disorder: Surface-based morphometry. J. Behav. Addict..

[18] Zvyagintsev M., Klasen M., Weber R., Sarkheil P., Esposito F., Mathiak K.A., Schwenzer M., Mathiak K. (2016). Violence-related content in video game may lead to functional connectivity changes in brain networks as revealed by fMRI-ICA in young men. Neuroscience.

[19] Kwak K.H., Hwang H.C., Kim S.M., Han D.H. (2020). Comparison of Behavioral Changes and Brain Activity between Adolescents with Internet Gaming Disorder and Student Pro-Gamers. Int. J. Environ. Res. Public Health.

[20] Dye M.W.G., Bavelier D. (2010). Differential development of visual attention skills in school-age children. Vision Res..

[21] Dye M.W.G., Green C.S., Bavelier D. (2009). The development of attention skills in action video game players. Neuropsychologia.

[22] Trick L.M., Jaspers-Fayer F., Sethi N. (2005). Multiple-object tracking in children: The “Catch the Spies” task. Cogn. Dev..

[23] Sessa M., Di Mauro G., Mascolo A., Rafaniello C., Sportiello L., Scavone C., Capuano A. (2018). Pillars and pitfalls of the new pharmacovigilance legislation: Consequences for the identification of adverse drug reactions deriving from abuse, misuse, overdose, occupational exposure, and medication errors. Front. Pharmacol..

[24] Liberati A., Altman D.G., Tetzlaff J., Mulrow C., Gøtzsche P.C., Ioannidis J.P.A., Clarke M., Devereaux P.J., Kleijnen J., Moher D. (2009). The PRISMA statement for reporting systematic reviews and meta-analyses of studies that evaluate health care interventions: Explanation and elaboration. J. Clin. Epidemiol..

[25] Operto F.F., Pastorino G.M.G., Marciano J., de Simone V., Volini A.P., Olivieri M., Buonaiuto R., Vetri L., Viggiano A., Coppola G. (2020). Digital Devices Use and Language Skills in Children between 8 and 36 Month. Brain Sci..

[26] Green C.S., Bavelier D. (2003). Action video game modifies visual selective attention. Nature.

[27] Feng J., Spence I., Pratt J. (2007). Playing an action video game reduces gender differences in spatial cognition. Psychol. Sci..

[28] Castel A.D., Pratt J., Drummond E. (2005). The effects of action video game experience on the time course of inhibition of return and the efficiency of visual search. Acta Psychol..

[29] Andrews G., Murphy K., Vanchevsky M.A. (2006). Does Video Game Playing Improve Executive Functioning?. Frontiers in: Cognitive Psychology.

[30] Colzato L.S., Van Leeuwen P.J.A., Van Den Wildenberg W., Hommel B. (2010). DOOM’d to switch: Superior cognitive flexibility in players of first person shooter games. Front. Psychol..

[31] Colzato L.S., van den Wildenberg W.P.M., Zmigrod S., Hommel B. (2013). Action video gaming and cognitive control: Playing first person shooter games is associated with improvement in working memory but not action inhibition. Psychol. Res..

[32] Powers K.L., Brooks P.J., Blumberg F.C. (2014). Evaluating the Specificity of Effects of Video Game Training. Learning by Playing: Video Gaming in Education.

[33] Spence I., Feng J. (2010). Video games and spatial cognition. Rev. Gen. Psychol..

[34] Stern Y., Blumen H.M., Rich L.W., Richards A., Herzberg G., Gopher D. (2011). Space Fortress game training and executive control in older adults: A pilot intervention. Aging Neuropsychol. Cogn..

[35] Maillot P., Perrot A., Hartley A. (2012). Effects of interactive physical-activity video-game training on physical and cognitive function in older adults. Psychol. Aging.

[36] McDermott A.F., Bavelier D., Green C.S. (2014). Memory abilities in action video game players. Comput. Hum. Behav..

[37] Basak C., Boot W.R., Voss M.W., Kramer A.F. (2008). Can training in a real-time strategy video game attenuate cognitive decline in older adults?. Psychol. Aging.

[38] Benoit J.J., Roudaia E., Johnson T., Love T., Faubert J. (2020). The neuropsychological profile of professional action video game players. PeerJ.

[39] Smirni D. (2020). The Raven’s Coloured Progressive Matrices in Healthy Children: A Qualitative Approach. Brain Sci..

[40] Bediou B., Adams D.M., Mayer R.E., Tipton E., Green C.S., Bavelier D. (2018). Meta-analysis of action video game impact on perceptual, attentional, and cognitive skills. Psychol. Bull..

[41] Homer B.D., Plass J.L., Raffaele C., Ober T.M., Ali A. (2018). Improving high school students’ executive functions through digital game play. Comput. Educ..

[42] Smirni D., Precenzano F., Magliulo R.M., Romano P., Bonifacio A., Gison G., Bitetti I., Terracciano M., Ruberto M., Sorrentino M. (2018). Inhibition, set-shifting and working memory in Global Developmental Delay preschool children. Life Span Disabil..

[43] Carotenuto M., Ruberto M., Fontana M.L., Catania A., Misuraca E., Precenzano F., Lanzara V., Messina G., Roccella M., Smirni D. (2019). Executive functioning in autism spectrum disorders: A case-control study in preschool children. Curr. Pediatr. Res..

[44] Oei A.C., Patterson M.D. (2014). Playing a puzzle video game with changing requirements improves executive functions. Comput. Hum. Behav..

[45] Oei A.C., Patterson M.D. (2013). Enhancing cognition with video games: A multiple game training study. PLoS ONE.

[46] Franceschini S., Gori S., Ruffino M., Viola S., Molteni M., Facoetti A. (2013). Action video games make dyslexic children read better. Curr. Biol..

[47] Franceschini S., Tancioni L., Lorenzoni M., Mattei F., Scardi M. (2019). An ecologically constrained procedure for sensitivity analysis of Artificial Neural Networks and other empirical models. PLoS ONE.

[48] Smirni D., Oliveri M., Turriziani P., Di Martino G., Smirni P. (2018). Benton visual form discrimination test in healthy children: Normative data and qualitative analysis. Neurol. Sci..

[49] Wang X., Goh D.H.-L. (2017). Video game acceptance: A meta-analysis of the extended technology acceptance model. Cyberpsychology Behav. Soc. Netw..

[50] Ferguson C.J. (2010). Blazing Angels or Resident Evil? Can Violent Video Games be a Force for Good?. Rev. Gen. Psychol..

[51] Bioulac S., Arfi L., Bouvard M.P. (2008). Attention deficit/hyperactivity disorder and video games: A comparative study of hyperactive and control children. Eur. Psychiatry.

[52] Chan P.A., Rabinowitz T. (2006). A cross-sectional analysis of video games and attention deficit hyperactivity disorder symptoms in adolescents. Ann. Gen. Psychiatry.

[53] Swing E.L., Gentile D.A., Anderson C.A., Walsh D.A. (2010). Television and video game exposure and the development of attention problems. Pediatrics.

[54] Christakis D.A., Zimmerman F.J., DiGiuseppe D.L., McCarty C.A. (2004). Early television exposure and subsequent attentional problems in children. Pediatrics.

[55] Landhuis C.E., Poulton R., Welch D., Hancox R.J. (2007). Does childhood television viewing lead to attention problems in adolescence? Results from a prospective longitudinal study. Pediatrics.

[56] Gentile D.A., Swing E.L., Lim C.G., Khoo A. (2012). Video game playing, attention problems, and impulsiveness: Evidence of bidirectional causality. Psychol. Pop. Media Cult..

[57] King D.L., Delfabbro P.H., Griffiths M.D., Gradisar M. (2011). Assessing clinical trials of Internet addiction treatment: A systematic review and CONSORT evaluation. Clin. Psychol. Rev..

[58] Bushman B.J. (2019). “Boom, Headshot!”: Violent first-person shooter (FPS) video games that reward headshots train individuals to aim for the head when shooting a realistic firearm. Aggress. Behav..

[59] Di Blasi M., Giardina A., Giordano C., Lo Coco G., Tosto C., Billieux J., Schimmenti A. (2019). Problematic video game use as an emotional coping strategy: Evidence from a sample of MMORPG gamers. J. Behav. Addict..

[60] Gee J.P. (2007). Good Video Games+ Good Learning: Collected Essays on Video Games, Learning, and Literacy.

[61] Greenfield P.M. (2009). Technology and Informal Education: What Is Taught, What Is Learned. Science.

[62] Griffiths M. (2000). Internet addiction-time to be taken seriously?. Addict. Res..

[63] Griffiths M. (2008). Internet and Video-Game Addiction. Adolescent Addiction.

[64] Weinstein A., Lejoyeux M. (2010). Internet addiction or excessive internet use. Am. J. Drug Alcohol Abuse.

[65] Griffiths M. (2005). Online therapy for addictive behaviors. CyberPsychology Behav..

[66] Griffiths M.D. (2010). Gambling addiction on the Internet. Internet Addiction: A Handbook and Guide to Evaluation and Treatment.

[67] Charlton J.P., Danforth I.D.W. (2007). Distinguishing addiction and high engagement in the context of online game playing. Comput. Hum. Behav..

[68] Charlton J.P., Danforth I.D.W. (2010). Validating the distinction between computer addiction and engagement: Online game playing and personality. Behav. Inf. Technol..

[69] Kuss D.J., Griffiths M.D., Pontes H.M. (2017). DSM-5 diagnosis of Internet Gaming Disorder: Some ways forward in overcoming issues and concerns in the gaming studies field: Response to the commentaries. J. Behav. Addict..

[70] Kardefelt-Winther D. (2014). Problematizing excessive online gaming and its psychological predictors. Comput. Hum. Behav..

[71] Przybylski A.K., Weinstein N., Murayama K. (2017). Internet gaming disorder: Investigating the clinical relevance of a new phenomenon. Am. J. Psychiatry.

[72] Kuss D.J., Griffiths M.D. (2012). Internet and gaming addiction: A systematic literature review of neuroimaging studies. Brain Sci..

[73] Thapa R., Nyamapfumba S. (2013). Neuroimaging of Addiction. J. Addict. Nurs..

[74] Hummer T.A. (2015). Media violence effects on brain development: What neuroimaging has revealed and what lies ahead. Am. Behav. Sci..

[75] Durlach P.J., Kring J.P., Bowens L.D. (2009). Effects of action video game experience on change detection. Mil. Psychol..

[76] Gentile D.A., Choo H., Liau A., Sim T., Li D., Fung D., Khoo A. (2011). Pathological video game use among youths: A two-year longitudinal study. Pediatrics.

[77] Ha J.H., Kim S.Y., Bae S.C., Bae S., Kim H., Sim M., Lyoo I.K., Cho S.C. (2007). Depression and Internet addiction in adolescents. Psychopathology.

[78] Morrison C.M., Gore H. (2010). The relationship between excessive Internet use and depression: A questionnaire-based study of 1,319 young people and adults. Psychopathology.

[79] Lam L.T., Peng Z.-W. (2010). Effect of pathological use of the internet on adolescent mental health: A prospective study. Arch. Pediatr. Adolesc. Med..

[80] Liau A.K., Choo H., Li D., Gentile D.A., Sim T., Khoo A. (2015). Pathological video-gaming among youth: A prospective study examining dynamic protective factors. Addict. Res. Theory.

[81] Greitemeyer T., Osswald S. (2011). Playing Prosocial Video Games Increases the Accessibility of Prosocial Thoughts. J. Soc. Psychol..

[82] Saleem M., Anderson C.A., Gentile D.A. (2012). Effects of Prosocial, Neutral, and Violent Video Games on Children’s Helpful and Hurtful Behaviors. Aggress. Behav..

[83] Gentile D.A., Anderson C.A., Yukawa S., Ihori N., Saleem M., Ming L.K., Shibuya A., Liau A.K., Khoo A., Bushman B.J. (2009). The effects of prosocial video games on prosocial behaviors: International evidence from correlational, longitudinal, and experimental studies. Personal. Soc. Psychol. Bull..

[84] Greitemeyer T., Osswald S. (2009). Prosocial video games reduce aggressive cognitions. J. Exp. Soc. Psychol..

[85] Sestir M.A., Bartholow B.D. (2010). Violent and nonviolent video games produce opposing effects on aggressive and prosocial outcomes. J. Exp. Soc. Psychol..

[86] Greitemeyer T., Traut-Mattausch E., Osswald S. (2012). How to ameliorate negative effects of violent video games on cooperation: Play it cooperatively in a team. Comput. Hum. Behav..

[87] Greitemeyer T., Mügge D.O. (2014). Video Games Do Affect Social Outcomes: A Meta-Analytic Review of the Effects of Violent and Prosocial Video Game Play. Personal. Soc. Psychol. Bull..

[88] Greitemeyer T., Osswald S., Brauer M. (2010). Playing prosocial video games increases empathy and decreases schadenfreude. Emotion.

[89] Prot S., Gentile D.A., Anderson C.A., Suzuki K., Swing E., Lim K.M., Horiuchi Y., Jelic M., Krahé B., Liuqing W. (2014). Long-Term Relations Among Prosocial-Media Use, Empathy, and Prosocial Behavior. Psychol. Sci..

[90] Harrington B., O’Connell M. (2016). Video games as virtual teachers: Prosocial video game use by children and adolescents from different socioeconomic groups is associated with increased empathy and prosocial behaviour. Comput. Hum. Behav..

[91] Buckley K.E., Anderson C.A., Vorderer P., Bryant J. (2006). A Theoretical Model of the Effects and Consequences of Playing Video Games. Playing Video Games—Motives, Responses, and Consequences.

[92] Gentile D.A., Reimer R.A., Nathanson A.I., Walsh D.A., Eisenmann J.C. (2014). Protective Effects of Parental Monitoring of Children’s Media Use: A Prospective Study. JAMA Pediatr..

[93] Bushman B.J., Huesmann L.R. (2006). Short-term and Long-term Effects of Violent Media on Aggression in Children and Adults. Arch. Pediatr. Adolesc. Med..

[94] Huesmann L.R., Kirwil L. (2007). Why Observing Violence Increases the Risk of Violent Behavior by the Observer. The Cambridge Handbook of Violent Behavior and Aggression.

[95] Maier J.A., Gentile D.A. (2012). Learning Aggression through the Media: Comparing Psychological and Communication Approaches. The psychology of Entertainment Media: Blurring the Lines between Entertainment and Persuasion.

[96] Corbett A.T., Koedinger K.R., Hadley W.H., Goodman P.S. (2001). Cognitive Tutors: From the Research Classroom to All Classrooms. Technology Enhanced Learning: Opportunities for Change.

[97] Kato P.M., Cole S.W., Bradlyn A.S., Pollock B.H. (2008). A Video Game Improves Behavioral Outcomes in Adolescents and Young Adults With Cancer: A Randomized Trial. Pediatrics.

[98] Smirni D., Carotenuto M., Precenzano F., Smirni P., Operto F.F., Marotta R., Roccella M. (2019). Memory performances and personality traits in mothers of children with obstructive sleep apnea syndrome. Psychol. Res. Behav. Manag..

[99] Lieberman D.A. (2001). Management of Chronic Pediatric Diseases with Interactive Health Games: Theory and Research Findings. J. Ambul. Care Manage..

[100] Lieberman D.A. (2006). What Can We Learn from Playing Interactive Games?. Playing Video Games: Motives, Responses, and Consequences.

[101] Jenkins H. (2006). No The war between effects and meaning: Rethinking the video game violence debate. Digital Generations: Children, Young People, and the New Media.

[102] Ferguson C.J., Kilburn J. (2010). Much ado about nothing: The misestimation and overinterpretation of violent video game effects in Eastern and Western nations: Comment on Anderson et al. (2010). Psychol. Bull..

[103] Sherry J.L. (2001). The Effects of Violent Video Games on Aggression: A Meta-Analysis. Hum. Commun. Res..

[104] Funk J.B., Baldacci H.B., Pasold T., Baumgardner J. (2004). Violence exposure in real-life, video games, television, movies, and the internet: Is there desensitization?. J. Adolesc..

[105] Anderson C.A., Gentile D.A., Buckley K.E., Anderson C.A., Gentile D.A., Buckley K.E. (2007). Violent Video Game Effects on Children and Adolescents: Theory, Research, and Public Policy. Violent Video Game Effects on Children and Adolescents: Theory, Research, and Public Policy.

[106] Konijn E.A., Nije Bijvank M., Bushman B.J. (2007). I wish I were a warrior: The role of wishful identification in the effects of violent video games on aggression in adolescent boys. Dev. Psychol..

[107] Gentile D.A., Lynch P.J., Linder J.R., Walsh D.A. (2004). The effects of violent video game habits on adolescent hostility, aggressive behaviors, and school performance. J. Adolesc..

[108] Anderson C.A., Dill K.E. (2000). Video games and aggressive thoughts, feelings, and behavior in the laboratory and in life. J. Pers. Soc. Psychol..

[109] Carnagey N.L., Anderson C.A. (2005). The effects of reward and punishment in violent video games on aggressive affect, cognition, and behavior. Psychol. Sci..

[110] Hopf W.H., Huber G.L., Weiß R.H. (2008). Media Violence and Youth Violence. J. Media Psychol..

[111] Ferguson C.J. (2007). The Good, The Bad and the Ugly: A Meta-analytic Review of Positive and Negative Effects of Violent Video Games. Psychiatr. Q..

[112] Bavelier D., Green C.S., Pouget A., Schrater P. (2012). Brain plasticity through the life span: Learning to learn and action video games. Annu. Rev. Neurosci..

[113] Green C.S., Bavelier D. (2008). Exercising your brain: A review of human brain plasticity and training-induced learning. Psychol. Aging.

[114] Gentile D.A., Bailey K., Bavelier D., Brockmyer J.F., Cash H., Coyne S.M., Doan A., Grant D.S., Green C.S., Griffiths M. (2017). Internet gaming disorder in children and adolescents. Pediatrics.

[115] Anderson C.A., Warburton W.A. (2012). The Impact of Violent Video Games: An Overview. Growing Up Fast and Furious: Reviewing the Impacts of Violent and Sexualised Media on Children.

[116] Gentile D.A. (2011). The multiple dimensions of video game effects. Child Dev. Perspect..

[117] Gentile D.A., Stone W. (2005). Violent video game effects on children and adolescents. A review of the literature. Minerva Pediatr..

[118] Stone W., Gentile D.A. The five dimensions of video game effects. Proceedings of the Annual Convention of the American Psychological Association.

[119] Khoo A., Gentile D.A. (2007). Problem Based Learning in the World of Games. Problem-Based Learning and e-Learning Breakthroughs.

[120] Anderson C.A., Gentile D.A., Dill K.E., Singer D.G., Singer J.L. (2012). Prosocial, Antisocial, and Other Effects of Recreational Video Games. Handbook of Children and the Media.

[121] Sharif I., Sargent J.D. (2006). Association Between Television, Movie, and Video Game Exposure and School Performance. Pediatrics.

[122] Berkey C.S., Rockett H.R.H., Field A.E., Gillman M.W., Frazier A.L., Camargo C.A., Colditz G.A. (2000). Activity, dietary intake, and weight changes in a longitudinal study of preadolescent and adolescent boys and girls. Pediatrics.

[123] Laurson K.R., Eisenmann J.C., Welk G.J., Wickel E.E., Gentile D.A., Walsh D.A. (2008). Combined influence of physical activity and screen time recommendations on childhood overweight. J. Pediatr..

[124] Vandewater E.A., Shim M., Caplovitz A.G. (2004). Linking obesity and activity level with children’s television and video game use. J. Adolesc..

[125] King D.L., Delfabbro P.H., Griffiths M.D. (2010). The role of structural characteristics in problem video game playing: A review. Cyberpsychology.

[126] Ferguson C.J., Beaver K.M. (2009). Natural born killers: The genetic origins of extreme violence. Aggress. Violent Behav..

[127] Ferguson C.J., Rueda S.M., Cruz A.M., Ferguson D.E., Fritz S., Smith S.M. (2008). Violent video games and aggression: Causal relationship or byproduct of family violence and intrinsic violence motivation?. Crim. Justice Behav..

[128] Anderson C.A., Bushman B.J. (2002). The effects of media violence on society. Science.

[129] Carnagey N.L., Anderson C.A. (2003). No Theory in the Study of Media Violence: The General Aggression Model. Media Violence and Children: A Complete Guide for Parents and Professionals.

[130] Gentile D.A., Groves C.L., Gentile J.R. (2014). The General Learning Model: Unveiling the Teaching Potential of Video Games. Learning by Playing: Video Gaming in Education.

[131] Hastings E.C., Karas T.L., Winsler A., Way E., Madigan A., Tyler S. (2009). Young Children’s Video/Computer Game Use: Relations with School Performance and Behavior. Issues Ment. Health Nurs..

[132] Pontes H.M., Schivinski B., Sindermann C., Li M., Becker B., Zhou M., Montag C. (2019). Measurement and conceptualization of Gaming Disorder according to the World Health Organization framework: The development of the Gaming Disorder Test. Int. J. Ment. Health Addict..

[133] Association A.P. (2013). Diagnostic and Statistical Manual of Mental Disorders (DSM-5®).

[134] King D.L., Delfabbro P.H. (2013). Video-gaming disorder and the DSM-5: Some further thoughts. Aust. N. Z. J. Psychiatry.

[135] Pontes H.M., Griffiths M.D. (2014). Internet addiction disorder and internet gaming disorder are not the same. J. Addict. Res. Ther..

[136] Kuss D.J., Griffiths M.D., Pontes H.M. (2017). Chaos and confusion in DSM-5 diagnosis of Internet Gaming Disorder: Issues, concerns, and recommendations for clarity in the field. J. Behav. Addict..

[137] Starcevic V., Aboujaoude E. (2017). Internet addiction: Reappraisal of an increasingly inadequate concept. CNS Spectr..

[138] Starcevic V. (2013). Is Internet addiction a useful concept?. Aust. N. Z. J. Psychiatry.

[139] Király O., Griffiths M.D., Urbán R., Farkas J., Kökönyei G., Elekes Z., Tamás D., Demetrovics Z. (2014). Problematic Internet use and problematic online gaming are not the same: Findings from a large nationally representative adolescent sample. Cyberpsychology Behav. Soc. Netw..

[140] Griffiths M. (2000). Does Internet and computer “addiction” exist? Some case study evidence. CyberPsychology Behav..

